# Shaping Ability and Debris Extrusion of New Rotary Nickel-Titanium Root Canal Instruments

**DOI:** 10.3390/ma14051063

**Published:** 2021-02-24

**Authors:** Sebastian Bürklein, David Donnermeyer, Tim Julian Hentschel, Edgar Schäfer

**Affiliations:** 1 Central Interdisciplinary Ambulance in the School of Dentistry, Westphalian Wilhelms-University, Albert-Schweitzer-Campus 1, Building W 30, 48149 Münster, Germany; sebastian.buerklein@ukmuenster.de; 2 Department of Periodontology and Operative Dentistry, Westphalian Wilhelms-University, Albert-Schweitzer-Campus 1, Building W 30, 48149 Münster, Germany; David.Donnermeyer@ukmuenster.de; 3 Private Practice, Zahnärztliche Gemeinschaftspraxis Die Kessels, Brüsseler Straße 11a, 53332 Bornheim, Germany; timotheush@web.de

**Keywords:** canal straightening, debris extrusion, endodontics, nickel-titanium, root canal preparation

## Abstract

The aim was to evaluate the canal straightening and the amount of apically extruded debris associated with five rotary nickel-titanium when preparing curved root canals. A total of 100 root canals in extracted human teeth (angles of curvatures 20°–30°; radii 5.9–13.5 mm) were divided into five groups (*n* = 20/group). The groups were balanced with respect to the angle and the radius of canal curvature. The root canals were prepared using conventional austenite 55-NiTi alloy instruments F360, F6 SkyTaper (both Komet, Lemgo, Germany), and the heat-treated NiTi Jizai, Silk-Complex and Silk-Standard instruments (all Mani, Tochigi, Japan) to an apical size 25. The amount of extruded debris was assessed with a micro balance. Statistical analysis was performed using Kruskal–Wallis test with Bonferroni correction at a significance level of *p* < 0.05. During canal preparation, neither instrument fractures nor procedural preparation errors were noticed. Regarding canal straightening, the use of Jizai instruments resulted in the significantly lowest straightening (*p* < 0.05), while no significant differences were obtained between all other groups (*p* > 0.05). Regarding the amount of apically extruded debris, no significant differences between all groups were obtained (*p* > 0.05). Within the limitations of this study, all instruments performed well, and especially the Jizai instruments showed an excellent shaping ability.

## 1. Introduction

Root canal treatment is indicated in the case of irreversible pulpitis and non-vital teeth with or without associated apical periodontitis. Despite numerous remarkable innovations in the field of endodontics during the last decade, root canal preparation of teeth with curved root canals still represents a challenge [1].

Although limited, the currently available evidence suggests that maintaining the original canal curvature during root canal preparation has a positive impact on the clinical outcome [2]. Engine-driven nickel-titanium (NiTi) instruments have been shown to cause less canal straightening during preparation of curved root canals than stainless steel hand instruments [3]. Coincidentally, the use of engine-driven NiTi instruments seems to also be associated with less apical debris extrusion during preparation than conventional stainless steel hand instruments [3,4].

The latter aspect, apical debris extrusion, is claimed to exert a clinically relevant impact on post-endodontic pain and thus on quality of life of patients after nonsurgical root canal treatment [5,6,7,8]. During the procedure of root canal preparation, pulp tissue remnants, dentine chips, microorganisms, and solutions used for canal irrigation may be extruded apically into the periradicular tissues [4,9]. In the periradicular tissues, extruded debris may cause expression of Substance P and Calcitonin gene-related peptide, which results in post-endodontic pain and in a reduced quality of life [9,10]. Evidence provided by two meta-analyses suggests that the kinematic of the instruments’ motion during root canal preparation has an impact on the intensity and duration of post-endodontic pain, whereby instruments used in a rotary motion caused less pain than those used in a reciprocation motion [11,12]. The prevalence of post-endodontic pain ranges between 40% (24 h after root canal preparation) and 12% (48 h after root canal preparation), respectively [13,14].

**Figure 1:**
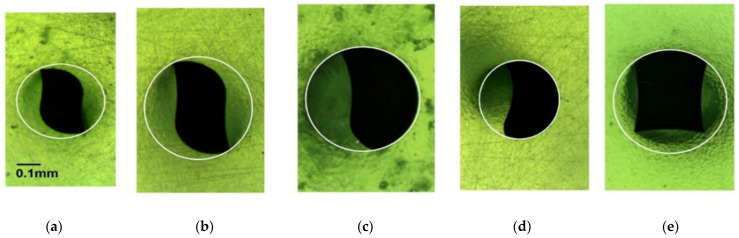
Cross-sectional shape of the instruments (size 25) used in this study at D5.(**a**) F360 (Taper .04); and (**b**) F6 SkyTaper (Taper .06): S-shaped; (**c**) Silk-Standard (Taper .06) and (**d**) Complex (Taper .04): Tear drop design with radial lands. (**e**) Jizai (Taper .04): Modified rectangle with radial lands and an offset mass of rotation.

Thus, from a clinical point of view, it is of interest to assess these relevant parameters, shaping ability in terms of canal straightening during preparation of curved root canals and apical extrusion of debris, of newly launched root canal instruments under laboratory conditions prior to performing clinical trials. Recently, new rotary NiTi instruments were launched by Mani (Tochigi, Japan), namely the Silk- and the Jizai-files. The Silk-files are available in two sequences of instruments, the Silk-Standard (instruments with a constant taper of 6%) and the Silk-Complex (instruments with a constant taper of 4%), whereas the Jizai-instruments have a constant taper of either 4% or 6%. All these instruments are made using a proprietary heat treatment and exhibit unique cross-sectional designs [15] (Figure 1). The design and the properties of these instruments have been described in detail previously [15].

Other, already established full rotary NiTi instruments having a constant taper of either 4% or 6% are the F360 and the F6 SkyTaper instruments (both Komet, Lemgo, Germany). The design features of both instruments have been described in detail previously [16,17]. In brief, the instruments of both systems are characterized by an S-shaped cross-section (Figure 1), are made of a conventional austenite 55-NiTi alloy, and possess a constant taper of 4% (F360) or 6% (F6 SkyTaper).

The null hypothesis tested was that there is no difference regarding (i) canal straightening and (ii) the amount of apically extruded debris when using F360, F6 SkyTaper, Jizai, Silk-Complex, and Silk-Standard instruments in curved root canals of extracted human teeth.

## 2. Materials and Methods

### 2.1. Specimens

The results of previous studies were considered [16,17,18] and power calculation using G*Power 3.1 (Heinrich Heine University, Düsseldorf, Germany) indicated that the sample size for each group should be at least 14 (f = 0.4; *α* = 0.05; 1-*β* = 0.8). As procedural errors (e.g., instrument fracture, canal blockage or ledges, patency cannot be achieved) may occur during canal preparation, 20 canals were used for each experimental group in order to compensate for potentially excluded samples.

A total of 100 extracted human mandibular molars were included. All had at least one curved root and root canal (usually the mesio-buccal canal). The apical region of these curved roots was inspected using a stereomicroscope under 20×-magnification (Expert DN, Müller Optronic, Erfurt, Germany) to ensure that the root had a single apical foramen. Diamond burs were used to create coronal access and apical patency of the canals was verified using a size 10 K-file (VDW, Munich, Germany). The inclusion criteria for all teeth were: Intact root apices, not yet root canal-treated and root canal width in the apical portion of the canal was smaller or approximately equivalent to size 15. Canal width was evaluated using silver points sizes 10 to 20 (VDW). Therefore, a size 15 silver point was inserted and if it was possible to place this point to full working length with a firm tug-back, it was assumed that the canal diameter in the apical portion was approximately equivalent to size 15. If no tug-back was noticed, the canal diameter was smaller than size 15. The same was done with a size 20 point. If this point could not be inserted to full working length, the teeth met the inclusion criterion.

Standardized radiographs were taken prior to root canal preparation with an instrument of size 15 inserted into the canal. Standardized radiographs with a constant source-to-film and object-to-film distance were then taken as previously described [16,17]. Based on these radiographs, the degree and the radius of canal curvature were assessed using a computerized digital image processing system [16,17]. The radii of all included teeth ranged between 5.9 mm and 13.5 mm, and the angles of curvature between 20.0° and 29.8° (Table 1). Moreover, the distance between the apex and the cemento-enamel junction was the third parameter to guarantee balanced experimental groups. The homogeneity of the five groups regarding these three parameters was evaluated using analysis of variance (ANOVA) and post-hoc Tukey-test (Table 1).

Once the preparation of the root canal was completed, another standardized radiograph with the final instrument inserted into the canal was taken in order to redetermine the angles of canal curvature. Canal straightening was defined as the difference between the initial angle of canal curvature and the angle of curvature after canal preparation. In each tooth, only one canal was prepared. The working length of all included canals was determined by subtracting 1 mm from the length where size K-file 15 was just visible at the apical foramen. Root canal preparation was performed by one operator, and a second operator analyzed canal straightening and amounts of extruded debris. The second examiner was blind regarding all experimental groups.

### 2.2. Collection of Debris

In order to collect the apically extruded debris and the irrigant used during preparation (bi-distilled water), each tooth was fixed in an individually prepared rubber plug, in accordance with the method described by Myers and Montgomery [19]. Both the extruded debris and the irrigant were collected in a pre-weighed receptor tube that was attached to the lower edge of the rubber plug. This assembly and the methodology used were described in detail previously [19,20]. In brief, during root canal preparation, the root apex of each tooth was suspended within the receptor tube while a second bottle was used to hold the device in a way that no contact to the collecting vial was possible. The bottle was vented with a 25 gauge needle alongside the rubber plug to equalize the pressure. During root canal preparation, the operator was unable to see the root apex as the bottle was covered with tape. Once the canal preparation was completed, each tooth was removed from the receptor tube, and bi-distilled water (1 mL) was used to wash off the debris adhering to the root surface into the receptor tube. In order to ensure that the moisture evaporated completely from the collected debris, the receptor tubes were kept in an incubator at 70 °C for 5 days. Thereafter, the tubes were weighed (accuracy ± 0.00001 g) in triplicate using an electronic balance (Sartorius Cubis, Göttingen, Germany) and the mean value was computed. The difference between the weight of the tube containing debris and the dry weight of the empty tube represented the weight of the collected debris.

### 2.3. Instrumentation Sequences

Prior to engine-driven preparation a glide path equivalent to ISO size 15 was established in all canals using stainless steel hand instruments (Pilot file; VDW). All root canal preparations were performed by one operator experienced in preparation with the different types of instruments. A randomly laid down sequence was used in order to avoid bias towards one group. A 6:1 reduction handpiece (Sirona, Bensheim, Germany) powered by a torque-limited electric motor (VDW.Silver Reciproc motor, VDW) was used for canal preparation. For each instrument the individual torque limit and rotational speed programmed in the file library of the motor were used. In accordance with the manufacturers’ instructions, all instruments were used in a picking, slow in-and-out motion. The preparation sequences were as follows:

F360: Instruments 20/.04 and thereafter 25/.04 were used to full working length. Rotational speed 300 rpm torque 1.8N cm.

F6 SkyTaper: Used as single-file system with 25/.06. Rotational speed 300 rpm torque 2.2 Ncm.

Jizai: Instrumentation sequence: 25/.04; 25/.06, both instruments to full working length. Rotational speed 500 rpm torque 3 Ncm.

Silk-Complex: Instrumentation sequence: 25/.08; 20/.04; 25/.04, the first instrument was used to 2/3 of the canal length, the other two to full working length. Rotational speed 500 rpm torque 3 Ncm.

Silk-Standard: Instrumentation sequence: 25/.08; 20/.06; 25/.06, the first instrument was used to 2/3 of the canal length, the other two to full working length. Rotational speed 500 rpm torque 3 Ncm.

Thus, the final preparation size was 25 for all groups, while the final taper was either 4% (F360 and Silk-Complex) or 6% (F6 SkyTaper, Jizai, and Silk-Standard). Four canals were prepared with one set of instruments.

As soon as the instrument had reached 2/3 of the canal length or full working length, respectively, it was removed. After each instrument or after three pecks (amplitude less than 3 mm) using the single-file F6 SkyTaper, the canals were irrigated with 2 mL of bi-distilled, and apical patency was verified using a size 10 K-file. The NaviTip 31ga needle (Ultradent, South Jordan, UT, USA) was used for irrigation and was inserted as deep as possible into the canal without resistance until 1 mm short of the predetermined working length.

### 2.4. Statistical Analysis

The Kolmogorov–Smirnov and Shapiro–Wilk tests were used to examine whether the data regarding canal straightening and amount of extruded debris were distributed normally. As the data were not distributed normally, further statistically analysis was performed using Kruskal–Wallis test with Bonferroni correction at a significance level of *p* < 0.05.

## 3. Results

During canal preparation, neither instrument fractures nor procedural preparation errors (e.g., perforations, ledges, blockages) occurred. Apical patency was maintained for all canals.

Regarding canal straightening, the use of Jizai instruments resulted in the significantly lowest straightening (*p* < 0.05), while no significant differences between all other groups were obtained (*p* > 0.05) (Table 2).

Regarding the amount of apically extruded debris, no significant differences between all groups were obtained (*p* > 0.05) (Table 3).

## 4. Discussion

In the present study, neither instrument fractures nor preparation errors occurred, and thus, all instruments were obviously safe to use. Moreover, regarding canal straightening, all instruments maintained the original canal curvature well (Table 2). The mean canal straightening ranged between 0.58° and 1.11°. F360 and F6 SkyTaper served as a kind of control group in this study (F360 for the instruments having a 4% taper and F6 SkyTaper for instruments having a 6% taper) to allow reliable classification of the new rotary NiTi instruments as both instruments have already been investigated using similar experimental conditions [16,17]. The obtained mean values for canal straightening were minorly lower in the present study than in previous investigations, which can be explained by the fact that in the previous studies, the mean canal curvature of the teeth was about 30° and in the present study merely about 25° [16,17]. On the whole, the obtained values for canal straightening of all instruments used in the present investigation are located in the low range already reported for similar rotary NiTi instruments [16,17,21] and from a clinical point of view, the values found for canal straightening seem to be neglectable. Surprisingly, the taper of the instruments exerted no relevant impact on the results, which is contradictory to previous results [1,17]. Usually, the greater the taper of an instrument, the lower its flexibility, and the more pronounced canal straightening is [1]. It can be speculated that either the innovative design features of the instruments tested outweighed the impact of the effect of greater tapers, or the canal curvatures used in this investigation were not severe enough to outpoint any impact of the taper. This aspect warrants further investigations using more severe and complex root canal curvatures.

The use of Jizai resulted in the significantly lowest canal straightening compared to all other instruments (*p* < 0.05; Table 2), which is in agreement with recently published findings [15]. This excellent shaping ability may be due to unique features of these instruments. Firstly, they are manufactured using a proprietary heat treatment [15]. It is well known that heat treatment of NiTi alloys may result in marked improvements of the properties of NiTi instruments [22], such as improved cyclic fatigue resistance [23]. Secondly, the Jizai instruments possess a unique asymmetrical cross-sectional design (Figure 1), resulting in an offset mass of rotation. The cross-sectional shape is characterized by radial lands at the shorter side of a modified rectangle. The impact of the cross-sectional shape of NiTi instruments on clinically relevant properties (such as bending properties and shaping ability) has often been proven previously [1,24,25].

The present results showed that all instruments caused apical extrusion of debris (Table 2). This finding is agreement with numerous previous studies as far as no preparation technique or any particular root canal instrument completely avoids debris extrusion [4]. In the present study, no significant differences were noted between the instruments tested (*p* > 0.05), and the obtained mean values were in the low range compared with previous studies using a variety of different NiTi instruments [9,18,19]. In general, the present results are corroborated by a recent meta-analysis on debris extrusion [9], and also indirectly by two meta-analyses focusing on the incidence of post-endodontic pain [11,12], as all these meta-analyses revealed that there is obviously a trend that rotary root canal preparation is associated with a reduced amount of apical debris extrusion. However, it must be confessed that direct comparisons of amounts of extruded debris are limited due to a major diversity of the experimental setups used. Clinical trials are now necessary to analyze whether the amounts of extruded debris found in the present study may have any clinical impact.

Several attempts have been made to standardize the experimental groups and the procedures as well as possible. Three parameters were defined (angle and radius of curvature; distance from apex to the cemento-enamel junction) to ensure well balanced experimental groups. According to the obtained *p*-values (0.993, 0.993, 0.876, respectively) homogenous groups resulted (Table 1). Moreover, the apical preparation size was the same (size 25) for all groups, and all instruments were used in a full rotary motion. Thus, any impact of different kinematics of the instruments on the obtained results was excluded. Furthermore, only instruments with a constant taper were used. This was done to minimize the effect of different variable tapers (e.g., regressive tapers as available for instance for WaveOne Gold (Dentsply Maillefer, Ballaigues, Switzerland) or Reciproc (VDW)) on the obtained results. The experimental setup used for determination of canal straightening is well established as several comparable investigations have been conducted previously using the same experimental conditions [16,17,21].

Moreover, the methodology used for assessment of extruded debris is in agreement with the laboratory model already described by Myers and Montgomery [19], and the limitations of the approach were discussed in detail in recent studies [19,20]. Apical patency was maintained throughout the entire root canal preparation procedures to ensure that the amount of apical extrusion was not limited. The most relevant limitation of the method used to determine apical debris extrusion is the fact that no physical back-pressure provided by the periapical tissues was simulated. Thus, gravity may have impelled the irrigant and debris out of the canal. Although these aspects must be taken into consideration when interpreting the obtained results, back-pressure of the periapical tissues was deliberately not simulated, as all described methods to mimic back-pressure suffer from other relevant shortcomings [26,27]. Floral foam has been recommended, but this material shows a remarkable absorption of irrigant and debris, thereby distorting the results considerably. Therefore, the used experimental setup still seems to be the best option to assess apical debris extrusion.

## 5. Conclusions

Based on the results of this laboratory study, the first null hypothesis was rejected, as the use of Mani Jizai resulted in significantly less canal straightening than all other instruments tested. However, the second null hypothesis was accepted, as no significant differences regarding the amount of apically extruded debris were found.

Within the limitations of the present study, all rotary instruments performed well and especially the Mani Jizai instruments showed an excellent shaping ability. Thus, randomized clinical studies are now required to evaluate whether the present results have a direct impact on the clinical outcome and the quality of life.

## Figures and Tables

**Table 1:** Parameters of the included curved root canals (*n* = 20 teeth per group; length = distance between apex to cemento-enamel junction; sd = standard deviation).

File	Angle (°)				Radius (mm)				Length (mm)			
File	Mean	sd	Min	Max	Mean	sd	Min	Max	Mean	sd	Min	Max
F360	25.3	3.03	20.5	29.8	8.8	1.67	5.9	12.4	14.2	1.49	11.5	16.7
F6	25.3	3.10	20.1	29.7	9.0	1.64	6.7	13.1	14.2	1.24	11.9	16.0
Jizai	25.0	3.07	20.1	29.5	8.9	1.91	5.9	12.5	13.9	1.55	10.4	16.4
Silk-Complex	25.1	3.11	20.0	29.6	9.0	1.42	7.0	12.5	14.1	1.34	11.0	17.0
Silk-Standard	25.2	3.11	20.1	29.7	9.0	2.00	6.0	13.5	14.3	1.67	11.6	17.0
*p*-value	0.993				0.993				0.876			

**Table 2:** Degree of canal straightening (°) after preparation with the different instruments (*n* = 20/group). Given are the means with standard deviation (sd), the minimum and maximum values, and the 95% confidence interval. Values with the same letters were not statistically different at *p* = 0.05.

Canal Straightening (°)						
File	mean	sd	min	max	95% Confidence Interval	
F360	1.00 ^b^	0.45	0.3	1.8	0.79	1.21
F6	1.11 ^b^	0.68	0.4	2.7	0.79	1.43
Jizai	0.58 ^a^	0.21	0.3	1.0	0.48	0.68
Silk-Complex	0.81 ^b^	0.27	0.3	1.2	0.68	0.93
Silk-Standard	0.93 ^b^	0.60	0.2	2.3	0.65	1.21
*p*-value	0.009					

**Table 3:** Amount of apically extruded debris (g) after preparation with the different instruments (*n* = 20/group). Given are the means with standard deviation (sd), the minimum and maximum values, and the 95% confidence interval. No statistically significant differences were obtained between the different groups.

Extruded Debris (g)						
File	Mean	sd	min	max	95% Confidence Interval	
F360	0.0018	0.00205	0.00	0.0072	0.0008	0.0027
F6	0.0020	0.00201	0.00	0.0065	0.0011	0.0030
Jizai	0.0011	0.00130	0.00	0.0043	0.0005	0.0017
Silk-Complex	0.0011	0.00170	0.00	0.0054	0.0003	0.0019
Silk-Standard	0.0015	0.00215	0.00	0.0068	0.0005	0.0025
*p*-value	0.32					
